# The Attitudes of Therapists and Physicians on the Use of Sex Robots in Sexual Therapy: Online Survey and Interview Study

**DOI:** 10.2196/13853

**Published:** 2019-08-20

**Authors:** Christiane Eichenberg, Marwa Khamis, Lisa Hübner

**Affiliations:** 1 Faculty of Medicine Sigmund Freud PrivatUniversität Vienna Austria

**Keywords:** robotics, sexual health, therapy

## Abstract

**Background:**

Various types of robots have already been successfully used in medical care, and the use of new technologies is also playing an increasing role in the area of sexuality. Sex robots are marketed as advanced sex toys and sex dolls with artificial intelligence. Only a few considerations about the therapeutic use of sex robots in sexual therapy are debated in expert discussions.

**Objective:**

The aim of this study was to conduct a first exploratory survey on the attitudes of sex therapists and physicians toward the therapeutic benefits of sex robots.

**Methods:**

This study comprised a quantitative online survey and a qualitative interview study. A self-constructed questionnaire was used to survey the general attitudes of sex therapists and physicians regarding the benefits of sex robots in therapy. The qualitative study was designed to gain in-depth insight into the participants’ beliefs and attitudes. Therefore, semistructured interviews were conducted. The quantitative data were evaluated by statistical analysis, and the interviews were transcribed and analyzed by using a grounded theory approach.

**Results:**

A total of 72 sex therapists and physicians completed our self-constructed questionnaire (response rate 15%, 72/480). Only a few respondents (11%, 8/72) said that the use of sex robots was not conceivable for them, and almost half of all therapists and physicians could imagine recommending sex robots in therapy (45%, 33/72). The attitude toward sex robots as a therapeutic tool was very heterogeneous, with gender (*P*=.006), age (*P*=.03), and occupational differences (*P*=.05); female therapists, older therapists, and psychologists (in contrast to physicians) were more critical toward the therapeutic use of sex robots. The analysis of the 5 interviews identified 3 high-level core themes that were representative of the participants’ responses: (1) the importance of the personal definition of sex robots for the assessment of their therapeutic benefits, (2) therapeutic benefits and dangers of sex robots, and (3) considerations on the quality of human-robot sexuality. Initial insights into the possible therapeutic use of sex robots in different disorders (eg, sexual dysfunction or pedophilia) and situations were gained from the perspective of sex therapists.

**Conclusions:**

The results of this study provide a first overview of the potential therapeutic use of sex robots. Moral, ethical, and treatment-related issues in this context are still unresolved and need to be further researched. We suggest integrating the topic into the training of sex therapists to form opinions beyond media images and to show therapy possibilities. Scientists engaged in sexual research should be involved in the development of sex robots to design robots with positive effects on sexual education, sexual therapy, sexual counseling, and sexual well-being for interested groups.

## Introduction

### Background

Robotics is an interdisciplinary field of research and practice, which is also relevant to sexuality because of the possibilities offered by human-machine interactions [1]. In the case of human-robot interaction, the emerging role of sex robots has piqued public interest. In the therapeutic debate about sex robots, it is important how psychologists and therapists define robot sex. The term technosexuality describes sexual activities that are combined with technology [2]. There are technosexual behaviors, such as internet pornography, that occur more frequently than others [3]. In the context of psychology, sexual activities with robots have mostly been described as objectophilia or robotic fetishism so far, whereby this definition is a rather pathologizing limitation. This is defined as a fetish attraction to humanoid or nonhumanoid robots, to people behaving like robots, or to people dressed in robot costumes [4]. In the most general and descriptive definition of robot sex, it can be defined as sexual use of the robot. This definition includes the use of special sex robots and the sexual use of other types of robots that are not specifically developed and marketed for sexual purposes [5].

Sex robots have triggered discussions in professional circles about robot design, social norms, and the status of human-robot sex in connection with human relationships as well as the possible benefits of sex robots [6]. For example, 1 benefit is in terms of using sex robots as a therapeutic tool in the treatment of sexual disorders. Different types of assistance and therapy robots have already been used for health care applications [7], for example, by people who are suffering from a stroke [8], dementia [9], autism [10], or physical disability [11]. A robotic assistant for health care applications can support users with training and rehabilitation programs that enable independent living. Although sex toys are used in sex therapy for the treatment of orgasm problems [12], there is no information about the opinion of sex therapists regarding sex robots as a tool in sex therapy. A robot designed for sex may have a different impact than other sex aids. Kerner [13] anecdotally reported that some sex therapists already have suggested a range of options that robots could help them with, including a variety of problems such as erectile dysfunction, ejaculatio praecox and social anxiety about having their first sexual encounter. In his book *Love and Sex with Robots,* David Levy [14] expressed a similar view when it comes to the potential psychosocial value of sex robots: “Many who would otherwise have become social misfits, social outcasts, or even worse will instead be better-balanced human beings.” Levy suggests that robotic sexual assistance contributes to health and well-being if it mitigates the exclusion of solo and partner sexuality associated with the impairment. Döring [15] adds that it would also be possible to use educational and therapeutic sex robots that allow certain exercise programs discreetly and without feelings of shame or guilt (eg, practice of safer sex techniques, treatment of orgasm disorders, and prevention of sexual assault). The statements quoted above are based on first considerations, but no therapists have been asked about their attitudes toward the therapeutic benefits of sex robots yet. First, we describe what types of sex robots are already available. Second, we discuss the existing controversy about sex robots, and finally, we summarize the current state of research.

### Types of Sex Robots

The success of sexual gratification dolls that can be defined as material representations of the human body for sexual use paved the way for the design of robots in the field of sexuality [16]. Although sex robots are still at a very early stage of development, the sex industry is already offering a variety of products that include some kind of artificial intelligence software. Sex robots already exist in female, male, and transgender versions with corresponding primary and secondary genitalia. Current sex robots, as well as sex dolls, are made of silicone rubber and advertised by manufacturers as *warm to the touch*. The appearance, such as eye color, hair, skin, and makeup, can be determined by the customer himself or herself. Prices for the sex robots range from about $5000 to $15,000. The sex robots marketed so far look like sex dolls but are capable of having conversations and display certain preprogrammed emotions or personalities. Some robots are equipped with allover body sensors so that they can react to touch. The response is sometimes dependent upon the chosen personality trait of the sex robot. For example, Roxxxy Gold (TrueCompanion) has preprogramed personalities, such as *Frigid Farrah* that gives the impression of reserved shyness. However, *Wild Wendy* is programmed as an outgoing and adventurous personality. Some sex robots offer a number of mobility features. Suzie Software and Harry Harddrive by Sex Bot Company must be manually maneuvered into a sexual position and are then able to simulate sexual movements. Roxxxy Gold is advertised by the manufacturer as being capable of displaying orgasms, although it is not clear whether this is through motion, sound, or both. Harmony (Realbotix) is also advertised as having the ability to simulate orgasm. The developers advertise the sex robot with the following capabilities: it has neck articulation, facial expression, moving eyes, and the ability to lip sync with spoken audio [17].

Robot sex also involves software robots without a materially embodied counterpart, in which the counterpart is represented virtually in an immersive virtual reality (VR) application. Immersive VR applications can also be combined with teledildonics. These are sex toys that offer haptic stimulation of male or female genitalia, synchronized with VR application [18].

### Controversy

In the international literature, the controversy about robot sex began 10 years ago, largely triggered by David Levy's monograph *Love and Sex with Robots* [14]. Since then, the topic sex robotics remains controversial; advocates interpret the development of sex robots as the next step in human-robot interaction and argue that robot sex will be a way to promote more openness in the context of sexuality. Sex robotics can be a health-promoting supplement and an expansion of partner sexuality. Another argument that emphasizes the benefits of sex robots is that people who cannot or do not want to commit (to a serious relationship) could benefit from it. The emotional attachment to a sex robot might be a helpful substitute that reduces loneliness and promotes well-being. The opponents’ side sees risks or, for example, conducts an ethical debate on whether human sexuality can be alienated in this way. A potential risk that critics often notice is that sex with robots could or will lead to social isolation [19]. The reasons vary as follows: spending time in a relationship with a robot could create an inability to form human friendships; real sexual relationships could become overwhelming as relations with robots are easier; robots do not meet the species-specific needs of humans; and sex robots could desensitize people to intimacy and empathy, which can only be developed by experiencing human interaction and consensual relationships.

Döring [5] explains that the polarization of the debate into pro and contrapositions (hype vs moral panic, utopian vs dystopian visions) is quite typical for dealing with technical innovations as there is no experience. Another problem with the public perception of sex robots is that the public is currently not well informed about robots in general. Sex robots are new, and information about them stem mainly from science fiction movies or books. Many of these science fiction stories focus on intimacy with robots, where robots (mostly female) are portrayed as sexual objects [20]. In a recent study, the media representations of intimate human-robot relationships were analyzed with the result that they reveal stereotypical gender roles, heteronormativity, and a focus on sexual versus emotional intimacy [21].

### Theoretical Framework

This study is based on the uses and gratifications approach. Studies following this approach investigate how recipients actively deal with media [22]. The aim of this concept is to find out the motives and utilization scenarios of the media usage preferences of the consumers. The users decide on the basis of their interests (content, formats, and aesthetics) and needs (eg, escape from reality, information, and entertainment) whether and what kind of media they prefer to use. The use of media thus depends on their expectations and the satisfaction of the needs the media offer [23]. The uses and gratifications approach is less a theory than a research strategy. Further developments such as the expectancy-value theory help to give this approach a stronger theoretical foundation. The expectancy-value theory deals with the relationship between attitude and behavior. It provides an explanation of how the subjective attitude toward an object affects the probability of performing certain actions. According to Fishbein’s model [24], it is believed that an attitude (A) toward an object (O) can be expressed in a function of beliefs (B) toward this object and the evaluations (E) of these expectations. According to this theory, there is a relationship between attitudes and the resulting behavior. Applied to our research project, this means that the expectation that sex robots possess a certain attribute, such as therapeutic potential, influences their use or recommendation. As sex robots is a new phenomenon, which is still in the development phase and experience reports have been missing so far, we wanted to survey the motives, possible use scenarios, and expectations of the therapeutic potential of therapists and physicians. This is of particular importance in the early phase of the development of sex robots so as to be able to contribute to this development from the perspective of the therapist.

### Current State of Research

A few studies about the acceptance of and attitudes toward sex robots already exist, whereby the study participants were not therapists or physicians, but the sample comprised people from the general population.

In 2016, Scheutz and Arnold [25] surveyed a sample of 100 US internet users (43% women, average age of 33 years) by using online questionnaires in the United States. They reported the first findings about the appropriateness and evaluation of sex robots with the result that many types of sex robot use were approved by the survey participants (eg, sex robots as an alternative to prostitution, sex robots for people with disabilities, sex robots to prevent violence), whereas only a few options or possibilities were rejected (eg, child sex robots). In their survey, subjects viewed sex with a sex robot as a kind of masturbation or using a vibrator than having sex with a human. In contrast to the agreement of the study participants on the definition of sex robots, the authors found relevant gender differences; women consistently rated each respective use and possible robotic form as less appropriate than men and were much less likely to consider a sex robot use in the future. A further survey study on attitudes toward sex robots among 203 German internet users (70% women, average age of 31 years) showed that 82.3% of respondents supported the use of sex robots, especially in cases of physical disabilities, instead of prostitution and as a way to live out certain sexual fantasies. Over 80% of the respondents could imagine the use of sex robots to treat a sexual problem (eg, ejaculatio praecox) and over 55% could imagine using sex robots in a therapeutic context [26].

A study by Szczuka and Krämer [27] compared men’s explicit and implicit evaluation of the (sexual) attractiveness of sex robots and women. The sample comprised 229 heterosexual men. It could be shown that social contacts, loneliness, fear of rejection, relationship status, and satisfaction with sexual life were not related to the evaluation of attractiveness. Instead, the authors found that the negative attitude toward robots was the main user characteristic that predicted the attractiveness ratings of sex robots. The study by Richards et al [28] with 133 participants also concluded that participants who generally viewed robots as negative, assessed the probability of having sex with a robot in the future as low.

The acceptance of sex robots among the general population is high. The same applies to the assessment of the potential benefits of sex robots for the treatment of sexual problems. As women and people with a generally negative attitude toward robots rate sex robots more negatively, it can be assumed that these groups of individuals may also have a lower acceptance of sex robots in the therapeutic context. The results lead us to the question whether sex therapists evaluate the use of sex robots similarly to the general population. The Foundation for Responsible Robotics [17] hypothesized that it is possible that the use of sex robots in some therapies could potentially help some people with sexual healing (eg, problems with sexual functioning or social anxiety). The lack of research and the simultaneous emerging interest in sex robots led us to the following questions, which we investigated in this study: What attitudes do sex therapists have toward the use of sex robots in the therapy of various sexual disorders? Do sex therapists consider sex robots as a therapeutic tool? Which patients could benefit from sex robots from the point of view of therapists? Do the therapists differ in their opinion on sex, age, education, personality, and affinity to technology?

## Methods

### Study Design

This was an exploratory pilot study to examine the attitudes toward and acceptance of sex robots in sex therapy by sex therapists and physicians in Germany, Austria, and Switzerland. A questionnaire was used for the quantitative survey, followed by a qualitative interview study with semistructured interviews to deepen the results.

### Ethics Approval

The study protocol was approved by the Ethics Committee of the Sigmund Freud University Vienna (approval number—electronic ID: LAWW6CYK@VV5BX86374).

### Survey

The quantitative data were collected with 3 questionnaires. The therapeutic acceptance of sex robots and the conditions under which the use of sex robots in sexual therapy appears acceptable were determined by using a self-developed questionnaire. This questionnaire was combined with 2 standardized questionnaires: The Questionnaire on technical affinity-attitude towards and handling of electronic devices (TA-EG) [29] to assess the affinity to technology and the NEO Five Factor Inventory [30] to gather personality traits of the sample and to test whether these factors had an influence on attitudes. The development of our questionnaire was based on the findings from the few existing studies on the acceptance of sex robots in the general population as described above. The self-developed questionnaire comprised 25 items and was divided into 3 parts. The first part included questions referring to participants’ characteristics such as age, employment, and education. In the second part, we provided the subjects with a short introductory text involving the definition of sex robots. As sex robots are new developments and the research field is so young, it can be assumed that the therapists themselves have little experience with robots. The participants were first asked about their knowledge of sex robots and subsequently about their personal attitudes. For example, we asked what use of sex robots would generally be conceivable and how therapists would categorize sexual activities with robots. With the questions in the third part, we wanted to assess the therapists’ attitudes toward the therapeutic potential of sex robots. A total of 2 standardized questionnaires were used to identify further possible factors that could influence the acceptance of sex robots. The self-constructed questionnaire was pretested with 5 participants. The few remarks were analyzed, and the instrument was revised regarding its practicability, comprehensibility, and completeness of item formulation.

With the TA-EG we wanted to learn more about the experiences and attitudes of the participants regarding technical devices. The subscales of TA-EG are enthusiasm for technology, subjective competence with technology, positive consequences of technology, and negative consequences of technology.

In the third and last part of the questionnaire, the NEO-FFI was used to collect personality traits to find out if they influence the attitudes of the participants and to learn more about the target group that is open to the therapeutic use of sex robots. The participants received 60 short statements describing themselves and were asked to evaluate the statements according to whether they applied to them or not. NEO-FFI factors include neuroticism, extraversion, openness to experience, tolerability, and conscientiousness.

### Interviews

Semistructured interviews were chosen for this purpose as they are considered as a valid and consistent method of data collection in qualitative research [31]. On the basis of the quantitative results, the interview guideline for the qualitative study was developed with the aim to further investigate open or controversially discussed aspects and determine possible therapeutic fields of implementation. As the quantitative survey showed that not all therapists had already heard of sex robots, the introductory question in the interview comprised what the interviewee knew about sex robots and where he or she obtained this knowledge from. A controversial result of the quantitative survey was the consideration of whether sex robots would be useful for the treatment of pedophile patients. On the basis of this result, we asked in the interview what the therapists’ attitudes toward this consideration was. Another result of the first survey that we wanted to address in the interviews was the tension between the conceivable use of sex robots in therapy on the one hand and ethical concerns on the other. In the interviews, we asked how these contradictions could be resolved. The complete interview guide can be found in Multimedia Appendix 1. The interviews followed a general-to-specific approach. Interviews were piloted before the use to make some adaptions if necessary.

### Sampling and Recruitment

For the quantitative survey, sex therapists and physicians were recruited through 4 professional associations: Institute for Sexual Therapy; German Society for Sexual Medicine, Sexual Therapy, and Sexual Science; Swiss Society of Sexology; and Austrian Society for Sexual Sciences. Cover letters were used to inform sex therapists and physicians about the survey, the implementation, the purpose of our investigation, and the exploitation of the results.

The theoretical sampling for the qualitative study was determined by the first results obtained from the quantitative data, which showed that sex therapists and physicians differ in their attitudes toward sex robots in gender, age, and education. Participants were sampled through Google searches. To obtain a broad spectrum of opinions in the interviews, female and male therapists with different ages and professional backgrounds were searched for. The information regarding age and education was found on the therapists’ homepage.

### Data Collection

The data for our online survey was collected using the Unipark software, which complies with all data protection regulations. The data collection took 4 weeks. A total of 480 therapists and physicians were contacted by email, which embedded a link to the online questionnaire. Of these, we received a total of 72 complete survey responses (response rate 15%). For the interviews, a total of 50 female and male therapists with medical, psychological, or social educational backgrounds of different ages were contacted by email. In total, 5 interviews were conducted by telephone and were digitally recorded. These ranged from 17 to 49 min, depending on the schedule of the participant and the number of issues they wanted to discuss.

### Coding and Data Analysis

Statistical analyses were conducted using SPSS 18. Descriptive statistics were used to evaluate most of the items. Statistical correlations were calculated using appropriate statistical test procedures (cross table, Spearman correlation, Wilcoxon-Mann-Whitney test, and binary logistic regression analysis). The open questions of the questionnaire were analyzed by content analysis. All interviews were transcribed verbatim. The transcripts were evaluated according to grounded theory analysis using Microsoft Office Word. The grounded theory is, among other methods, one that is suitable for research that seeks to discover something new [32]. Our choice of using grounded theory lies in its capacity to discover participants’ main concerns to consolidate the results of the quantitative survey. The analysis begins with the process of open coding. Open codes are, for example, certain words that occur recurrently in the data. The open codes are used to search for differences, similarities, behavioral patterns, etc, with the aim of forming categories [33]. The data were read and reviewed, and then a coding framework was developed to summarize the grouping of emerging issues from the data into core issues. The subsequent axial coding was about identifying the core phenomenon, causal conditions, resulting strategies (ie, related actions and interactions), context, and consequences. The collected data from each interview were further compared with each other and with the data from the other interviews to find similarities, repetitions, or differences in the emerging issues. Additional grouping codes were added as new topics emerged during the comparison process. Finally, all findings were extensively rereviewed by all authors to validate the data and to gain a high-level understanding of the collected information that would help to identify potential participant attitudes. The data collected in core themes are most relevant for the purposes of this study and are presented in the results.

### Sample

#### Online Survey

The participants of the quantitative survey were all members of sexual associations in Germany, Austria, and Switzerland. A total of 72 sex therapists and physicians completed the self-constructed questionnaire. Table 1 summarizes the description of the sample according to the variables: gender, age, relationship status, education, and information on therapeutic work.

#### Interview Study

All interviewed therapists were very well informed about sex robots, had technical knowledge, attended advanced training courses on the subject, and were familiar with the various application areas of sex robots*.* In total, 3 women and 2 men were interviewed for the qualitative study. Table 2 below gives information about the participants.

**Table 1:** Sample description of quantitative study (N=72).

Variables		Statistics
**Gender, n (%)**		
	Male	27 (38)
	Female	45 (62)
**Age (years)**		
	Range	32-80
	Mean (SD)	51 (9.6)
**Relationship status, n (%)**		
	Married	38 (52)
	Long-term relationship	19 (26)
	Divorced	7 (9)
	Single	5 (6)
	Other relationship	3 (4)
**Main training (multiple answers were possible), n (%)**		
	Sexual therapeutic education	64 (89)
	Sexual therapists	53 (83)
	Sexual physicians	11 (17)
	Psychotherapists	25 (35)
	Psychologists	15 (21)
	Physicians	15 (21)
**Psychotherapeutic method (multiple answers were possible), n (%)**		
	Psychodynamic therapy	17 (68)
	Systemic therapy	10 (40)
	Cognitive behavioral therapy	8 (38)
	Gestalt therapy	7 (28)
**Therapeutic settings (multiple answers were possible), n (%)**		
	Individual therapy	55 (87)
	Couple therapy	50 (79)
	Outpatient setting	42 (66)
	Group therapy	10 (15)
**Mainly treated disorders (multiple answers were possible), n (%)**		
	Orgasmic disorders	56 (78)
	Erectile dysfunction	51 (71)
	Mental disorders	28 (40)

**Table 2:** Sample description of interview study.

Person	Gender	Age (years)	Profession
1	Male	71	Sex therapist, psychologist
2	Female	54	Sex therapist, physician
3	Female	50	Sex therapist
4	Male	53	Sex therapist
5	Female	77	Psychotherapist, psychologists, active in the academic field of sexual sciences

## Results

### Online Survey

#### Previous Knowledge of Sex Robots

To find out how well known the topic of sex robots is among therapists and physicians, we asked them whether they had already heard or read about sex robots or seen something about them. A total of 56 participants of the sample (77%, 56/72) had already heard of sex robots. The majority received their information via the internet (51%, 29/56). Approximately one-third (32%, 18/56) said they had heard about them at a further training course and another 30% (17/56) reported to have read about sex robots in a scientific journal.

#### General Attitudes Toward Sex Robots

The question “How positive do you generally rate the existence of sex robots?” was analyzed on the basis of frequencies. Participants were allowed to indicate their percentage score based on an interval from 0% to 100%. The mean was 32% (SD 29.27). Accordingly, the existence of sex robots was not rated as very positive. The majority of respondents believed that sex with a robot could not replace sex with a human being (90%, 65/72). More than half of the participants would define sex with a robot as masturbation (58%, 42/72).

The participants of this study were asked which use of sex robots would be imaginable for them. To be able to assess this, we provided various situations, motives, and robot use patterns, whereby the interviewees were able to indicate which use of sex robots would be conceivable. The responses (Table 3) showed that the sample (N=72) had different attitudes toward the general use of sex robots. Only 8 out of 72 respondents (11%, 8/72) stated that the use of sex robots was not conceivable for them. The majority of participants stated that they could imagine the use of sex robots for physically disabled people (65%, 47/72) and for living out certain sexual fantasies (61%, 44/72). However, the idea that sex robots can help to experience a trusting sexual relationship got the least approval by survey participants (5%, 4/72). In the open response category, 2 persons indicated that any use of sex robots would be imaginable, and 1 person suggested the use of sex robots in connection with sexuality for older people.

#### Potential of Sex Robots as a Therapeutic Tool

One part of the questionnaire related to the potential of sex robots as a tool in sex therapy as well as to the idea of recommending sex robots in the role of a practitioner (eg, “If you think about your practice and your experience: which use of sex robots would be conceivable for you as a practitioner for your patients?”). The participants were asked about their imaginable use of sex robots, judging by their work and experiences as a therapist.

At the time of the survey, none of the respondents had already recommended the use of sex robots to a patient. Some sex therapists recommended the use of sex toys to their patients, for example, as a couple exercise at home. Other therapists completely disagreed and declined such recommendations. Overall, almost half of all respondents (45%, 33/72) could imagine recommending sex robots in therapy. The entire sample was asked about therapeutic situations in which they would consider the use of sex robots conceivable. The answers to the question about imaginable situations for the general use of sex robots showed a similar frequency distribution as the question about imaginable situations from the point of view of the practitioner. Most of the respondents could imagine the use for people with physical disabilities (61%, 44/72), to live out sexual fantasies (48%, 35/72), and for people living in isolated environments, for example, prisons (44%, 32/72). In comparison with the question regarding which use of sex robots would be generally conceivable for the participants, it was noticeable that the respondents were less in favor of recommending sex robots in a therapeutic setting.

#### Use in Various Sexual Disorders

In the questionnaire, we asked sex therapists and physicians which diagnoses (eg, based on 10th revision of the International Statistical Classification of Diseases and Related Health Problems, ICD) they regarded as suitable for the use of sex robots and for which purposes they would recommend a sex robot to support therapy. To answer this question, diagnoses from the ICD-10 and possible problems were mentioned. One-third (33%, 24/72) rejected any use for their patients. Only 19% (14/72) could imagine using sex robots for patients who wanted to improve their sexual relationship. The most frequent use was conceivable in *patients with social anxiety that prevents a sexual life* (50%, 36/72), for patients who do not have a partner and still want to have a sex life without having to resort to prostitution or fleeting acquaintances (50%, 36/72), and in patients with *ejaculatio praecox* (47%, 34/72). Table 4 gives a detailed overview of the results.

In addition, the participants were asked via open questions for which other patient groups they considered the use of sex robots as useful. This option had been used 25 times. Overall, 2 therapists added that they could see benefits for older people and patients suffering from dementia. Furthermore, 4 people mentioned *pedophile patients* in the open response category. Another 4 emphasized the use in the context of social anxieties and 7 persons stated that the use of sex robots had to be decided on an individual basis. The remaining 8 people named other diagnoses, such as autism or ejaculatio praecox.

**Table 3:** Attitudes toward different uses of sex robots (N=72).

Imaginable use of sex robots	Statistics, n (%)
For physically handicapped persons	47 (65)
To be able to live out certain sexual fantasies	44 (61)
In isolated environments, for example, prisons, space stations, etc	36 (50)
To temporarily replace a human sexual partner	34 (47)
To be able to experience a sexual relationship when, for certain reasons, a sexual relationship with a person cannot arise	34 (47)
Instead of prostitution	31 (43)
To improve general psychological well-being	30 (41)
As a sex toy in the relationship	30 (41)
Out of sexual interest	30 (41)
To discover sexual pleasure for yourself (again)	28 (38)
To learn sexual practices	27 (37)
As a remedy for loneliness	24 (33)
Instead of cheating on the partner	23 (31)
For pornographic movies	23 (31)
To make forms of sexual harassment/sexual violence more tangible for training and prevention purposes	23 (31)
Out of technological interest	22 (30)
To expand your own sexual practices	20 (27)
To have sex regularly	19 (26)
As a remedy for boredom	18 (25)
For mixed group sex with humans and sex robots	14 (19)
To practice intimacy with someone else	12 (16)
To minimize the risk of sexually transmitted diseases	12 (16)
To build a sexual relationship	11 (15)
To facilitate the practice of religious/spiritual abstinence	8 (11)
To permanently replace a human sexual partner	5 (6)
To experience a trustful sexual relationship	4 (5)
No use at all would be conceivable	8 (11)

**Table 4:** Evaluation of therapeutic use in different diagnoses and situations (N=72).

Diagnoses and situations of sex robot use in therapy	Statistics, n (%)
For patients with social anxiety	36 (50)
For people who do not have a partner and still want to lead a sex life without having to resort to prostitution or fleeting acquaintances	36 (50)
Ejaculatio praecox	34 (47)
Erectile dysfunction	29 (40)
Psychoeducation	28 (38)
Orgasm disorders	27 (37)
Vaginismus	23 (31)
Paraphilias	22 (30)
Sexual aversion	17 (23)
Frigidity	16 (22)
Dyspareunia	15 (20)
Patients who want to improve their sexual relationship with their partner	14 (19)
Sexual maturity crisis	14 (19)
Sex addiction	11 (15)
Gender identity disorders	9 (12)
Not at all	24 (33)

#### Future Use of Sex Robots in Therapy

In addition, the respondents of the questionnaire were asked future-oriented questions such as “How likely do you think you’ll be using sex robots in therapy within the next year/the next 5 years/the next 25 years?” For this question, the participants could choose an answer on 4 scales, ranging from very probable to very unlikely. It was found that 90% (64/72) of the therapists thought that the use of sex robots in therapy within the next year was very unlikely or unlikely. Only 68% (49/72) thought that they were very unlikely or unlikely to recommend a sex robot in the next 5 years, whereas 32% (18/2) thought they would consider it. When asked what would happen in the next 25 years, therapists were more likely to consider a recommendation. Only 38% (27/72) thought that a recommendation would be (very) unlikely, whereas 62% (45/72) thought it would be highly likely.

The content analysis of the open question whether sex robots will change sexuality showed that the answers could be divided into 3 categories. Some therapists and physicians emphasized positive changes, such as the expansion of sexuality and therapy options. Others noted negative effects, such as the loss and replacement of real human relationships, and some statements can be described as neutral. In total, 46 people gave open answers with 49 units of sense being indicated. Table 5 gives an overview of the results.

**Table 5:** Future changes in sexuality caused by sex robots as predicted by surveyed therapists.

Category	Example answers	Number of units of senses
Positive aspects	“Expansion of therapy options”; “Relieve tension”; “Enhancing sexual possibilities”; “Experience sexual pleasure and emotional attention”	12
Neutral aspects	“Variant of sexuality”; “A new sex toy”; “Comparable to the internet”; “Another option”	17
Negative aspects	“Sensuality gets lost”; “Build pressure”; “Dehumanization of sexuality”; “Prohibiting practicing with partner”	20

#### Ethical Problems

In the questionnaire, we also asked therapists to indicate whether using sex robots could lead to ethical issues, with 62% (45/72) of the sample answering this question in the affirmative. To obtain detailed information on possible ethical problems, an open question was asked. In total, 30 persons gave open answers with 34 units of sense being indicated. The content analysis of the open question revealed 5 categories. The first category *dehumanization* described how people are dehumanized by the comparability with robots. However, spending time with sex robots could make people no longer distinguish between humans and robots. The second category *violence* collected responses expressing concerns that the use of sex robots could promote sexual violence. The third category *neglect of interpersonal relationships* referred to statements expressing concerns that interpersonal relationships were disregarded through the use of sex robots. The fourth category was designated as *narcissistic disorders/selfishness*. Some statements related to the concern that selfishness might increase and were summarized in this category. In the last category, we summarized answers that do not address an ethical problem, such as *promotion of sex addiction*, which imply mental health topics. Isolated statements that did not fit into any of the categories were considerations about the (ethic) rights of robots, especially when artificial intelligence is so advanced that it resembles human consciousness. It was also stated that sex robots could lead to the misconception that sexual therapy was no longer needed. Table 6 gives an overview of the categories of ethical problems that have been identified.

**Table 6:** Ethical problems as predicted by surveyed therapists.

Category	Example answers	Number of units of senses
Dehumanization	“No more distinction between robots and human”; “Dehumanization”; “Confusion between human and machine”	10
Violence	“Performing trial offences”; “Risk of crossing borders in sexual contact with people”; “Sexual violence”; “Glorification of sexuality with children”	7
Neglect of interpersonal relationships	“Partner replacement, that is, no social relationship is established”; “Lack of understanding and flexibility in interpersonal relationships”; “Alienation from oneself, other people, and the world”	6
Narcissistic disorders/selfishness	“The tendency to use other people to satisfy one’s own needs will increase”; “More narcissistic disorders”	4
Nonethical problems mentioned	“Sex addiction”; “Addiction development”; “Strengthening of sexual dysfunction”	5

#### Relationship Between Attitude and Sociodemographic Data

We also tested whether the therapists’ and physicians’ attitudes differ with regard to gender, age, and profession. The examined attitudes are the conceivable use of sex robots in certain situations, as well as the ability to recommend a sex robot as a practitioner in certain situations and the willingness to recommend a sex robot for certain diagnoses or sexual disorders. Only significant results are presented below.

##### Relationship Between Attitude and Gender

In the quantitative survey, male and female therapists differed in their attitudes toward sex robots. There was a significant difference between women and men regarding the variable *sex with a robot has therapeutic potential* (χ²_1_=7,5 N=72; *Phi*=−0.324; *P*=.006), to the effect that men affirmed this variable more often than women. In addition to the question of ethical problems, there was a gender difference, namely, female therapists more often assumed ethical problems than male therapists (χ²_1_=6,0, N=72; *Phi*=0.289; *P*=.01).

##### Relationship Between Attitude and Age

There were differences in attitudes toward sex robots in general and also in therapeutic practice between younger and older therapists. There was a significant difference in age with regard to the imaginability of the general use of sex robots (χ²_2_=6.4, N=72; *P*=.03). The post hoc tests in pairs showed that younger therapists (n=30, aged 32-50 years) and middle-aged therapists (n=31, aged 51-60 years) of this sample differed from older therapists (n=11, aged 61-80 years) with regard to the conceivability of a general use of sex robotics. Younger therapists (*U*=84, *Z*=−2.39, *P*=.02) and middle-aged therapists (*U*=90, *Z*=−2.31, *P*=.02) could imagine the use more frequently than older therapists. A significant age difference could also be observed with regard to the recommendation of sex robots for certain diagnoses (χ²_2_=7.2, N=72; *P*=.03). Younger therapists (*U*=96, *Z*=−2.04, *P*=.04) and middle-aged therapists (*U*=99, *Z*=−2.04, *P*=.04) were more open regarding the recommendation of sex robots.

##### Relationship Between Attitude and Education

The therapists also differed in their attitudes toward sex robots with regard to their profession. There was a clear correlation between the profession and the idea of recommending sex robots to patients. A medium positive correlation was found between the recommendation of sex robots in certain situations and the profession *physician* at rho=0.296 and *P*=.01, whereas the profession of sex therapist correlated negatively with this variable at rho=−0.233 and *P*=.049. There was another significant difference regarding *sex with a robot has therapeutic potential*. Physicians (χ²_1_=4.3, N=72; *Phi*=0.245; *P*=.03) confirmed this variable much more frequently than others. A significant difference could also be observed with regard to ethical problems. The variable *could the use of sex robots lead to ethical problems* was denied by physicians more frequently than by other occupational groups (χ²_1_=4.74, N=72; *Phi*=0.249; *P*=.03).

#### Relationship of Attitude With Affinity to Technology and Personality Traits

A large part of the sample (88%, 63/72) also completed the TA-EG and the NEO-FFI. Binary logistic regressions were calculated to check whether the general attitude toward sex robots as well as the assessment of the therapeutic potential could be predicted by the factors of NEO-FFI and the scales of TA-EG. However, no relationship could be established between these aspects and the attitudes toward the therapeutic use of sex robots.

##### Definition of Sex Robots: Consumer Products Versus Therapeutic Tools

One result of the qualitative study is that the subjective definition of sex robots influences the evaluation of them as a therapeutic tool. Two different positions became apparent. One definition understands sex robots as a consumer product that is only used for physical satisfaction. “As consumer goods, sex robots are not a good development for sexuality and interpersonal relationships.” According to the same therapist, sex robots differ from other technical aids (eg, vibrators). Furthermore, 2 people compared sex robots with the *artificial world* of pornography: “I think of sex robots just as critically as sex for sale or pornography—like anything that suggests an artificial world.” Another therapist explains “Sex robots interfere with the development of pornography, sex toys, and internet-related sexuality.” With regard to this definition, possible problems such as internet sex addiction are mentioned above all. Therapists who defined sex robots primarily as a consumer product assessed their existence and use as negative. In contrast, therapists who defined sex robots as technical or therapeutic devices assessed their existence more positively. One therapist explains “It is important to distinguish between the social use of sex robots, i.e. consumer goods, and sex robots as therapeutic tools in order to identify their therapeutic benefits.” Therapists who defined sex robots as therapeutic tools described concrete ideas of how they should look like and work to actually be suitable for therapy. The skin of the robot was most frequently addressed in this context. A therapist described why skin sensation is important: “We know that the bonding hormone oxytocin is produced through skin contact between humans. The question would be if this also works for robots?” Another important point is that the robot body should resemble the human body. For therapists, this means that the robot body portrays an *imperfect* design to convey a healthy body image. The question “What kind of image of a woman is created by such a robot?” is also related to considerations about the optics of the robot. Another important issue was that sex robots should not be *conceived as slaves* but should have their own desires and needs. In addition, they should be able to express those needs, feelings, or desires.

##### Therapeutic Benefits and Dangers of Sex Robots

Many thoughts about the therapeutic benefits and dangers of sex robots from the point of view of sex therapists could be collected. Therapists who saw therapeutic benefits in sex robots also expressed ambivalent feelings toward them: “Even though I want to be open for this development, I have ambivalent feelings, for example, when I think of the loss of social skills as a possible consequence.”

All therapists described the concern that the use of sex robots could lead to loneliness, *further autonomization of instincts*, and *loss of social skills and loss of interpersonal relationships*. These concerns were based on the therapists’ experiences with the negative effects of excessive pornography consumption and on the assumption that *sex robots are part of this development*. The results of the quantitative survey, which showed the strongest agreement among therapists for the use of sex robots in physically handicapped people, in isolated environments, and instead of prostitution, could also be confirmed in the qualitative study. Even therapists who could not imagine any therapeutic use saw a general benefit of sex robots in these areas: “The only thing I could imagine is a benefit for physically handicapped people or even instead of prostitution so that fewer women have to suffer.” The therapeutic benefit of sex robots was discussed in the context of different disorders.

###### Patients With Deviant Sexual Behavior

The use of sex robots for patients with deviant sexual behavior was discussed by all therapists. Sex robots could have the potential to reduce the sex drive of certain sexually active persons within the framework of therapy. “Whenever sexuality becomes dangerous, the use of sex robots is worth considering if it can protect a real human life.” Therapists mention the use of sex robots in the context of sexual violence or rape and in the context of pedophile patients, with the strongest contrast of opinions being seen here. What seems important here is that pedophile patients must be treated differently. For some, an impulse control disorder is predominant, whereas others may be traumatized. Therapists point out that the benefits of sex robots must be decided individually for each specific case: “Pedophile patients are not all the same and it has to be decided here quite individually which patient could benefit from it.” For some patients, it could be an opportunity to live out their sexuality with a sex robot. Then, they could discuss in therapy which fantasies were behind it (eg, not being able to cope with an adult). For some patients, the use of sex robots could be a kind of substitute. For others, the stimuli for the abuse of children might intensify. A therapist pointed out the following: “It should be considered that the neuronal connection could be intensified by living out the fantasies with child sex robots in the patients’ brain.” Another therapist assumed that the abuse would be intensified by the use of child sex robots and underlined “that the production of child sex robots is generally immoral.” In contrast to this, another therapist argued that the patient’s thoughts, for example during masturbation, could also lower the barrier to committing a crime and that prohibitions—important as they may be—do not necessarily reduce the number of criminal offences, but rather provide an additional attraction for many patients. The therapist argued as follows: “If a child can be protected, then it makes sense to torture a doll instead.” Another therapist addressed one’s own fear of *triggering* something in the patient by recommending sex robots to pedophile patients. The responsibility of the therapist was also addressed. Does a therapist want to take responsibility for recommending sex robots, even if the therapy with a sex robot turns out to be dangerous and the patient becomes violent? Finally, several therapists addressed the need for further research in this field: “It would need more applied research in this particular area to actually generate therapeutic benefits for pedophile patients.”

###### Patients With Contact Disorders

The use of sex robots for people with contact disorders—the emotional interaction with another person is limited because of social anxiety—was also controversially evaluated. Those who completely reject the use of sex robots in therapy explained that sex robots are only a solution for loneliness for a short time. The use of sex robots could be seen as a kind of *substitute* or even *escape*, as those patients often experience so much fear to deal with a *real counterpart*. However, the use of sex robots could at least lead to a *1-dimensional satisfaction* or, depending on how sex robots are designed, could even help to practice social behavior and communication about sexuality, desires, needs, and borders.

###### Disorders of Female Sexuality

Some therapists discussed the use of sex robots in the context of the patient’s gender, by referring to supposed differences between female and male sexuality, whereby male sexuality was described as more *animal instinctive*. Although all therapists could imagine the use of sex robots in therapy rather for male patients, we can also describe some application areas for female patients. In the context of female sexuality, the therapeutic benefits of sex robots regarding desire and orgasm disorders, vaginismus, and traumatic experiences were discussed: “I could imagine that traumatized women who can ride on a sex robot, for example, and who can do so without fear of being overwhelmed by their sex partner, can benefit from this experience and successively reduce their fears, or that penetration will perhaps only become possible again in the first place.” Through a penetration-capable sex robot, women with traumatic experiences, such as sexual violence/rape, could reduce their fears, approach their own sexuality again, and regain access to their own bodies.

##### Quality of Human-Robot Sexuality

This category collects considerations relating to the quality of human-robot sexuality. The therapists explained what they understood by healthy sexuality and how sexuality had already changed because of pornography, the internet, and technical equipment. In this context, reflections were made on the question of how sexuality will be further changed by new developments such as sex robots. For therapists, sexuality has something to do with desire, eroticism, and communication. A therapist described the core of sexuality as something mental, but the physical part of it remains unmentioned here: “The core of the sexual event is the mental encounter.” Sexuality must be negotiated, and it must go beyond the *technical satisfaction* that a sex robot can offer, “it is also about warmth, appreciation, and respect.” Therapists postulated that the quality of a human relationship can never be achieved with a robot that, despite artificial intelligence, comprises inanimate material: “I’m sure a robot can never replace a real human relationship.” Some therapists are critical about the future use of sex robots and fear that the quality of human relationships and sexuality could suffer from this development. However, others are certain that an *emotional peak* that humans strive for can never be achieved by robot sex. There is agreement that sex robots will play a growing role in sexuality and sexual therapy in the future: “I am sure that sex robots will be an important topic in the future—this development will come.” All therapists argued that sex robots should not be seen as a substitute for human relationships and sexuality. Nevertheless, some therapists also see the potential of sex robots for sexuality. Sex robots could increase sexual satisfaction and provide an opportunity for more experimentation and sexual imagination.

### Interview Study

Core Categories. The aim of the qualitative study was to gain more detailed insights into possible therapeutic uses of sex robots for different types of patients. Overall, 3 categories were generated as follows: (1) definition of sex robots: consumer goods versus therapeutic aids, (2) benefits and dangers of sex robots, and (3) quality of robot sexuality.

## Discussion

### Principal Findings

#### Summary Discussion

Following the uses and gratifications approach, this study is one of the first ones to systematically explore motives, possible application scenarios, and attitudes of sex therapists toward sex robots as a tool in sexual therapy. The presented results about the possible therapeutic use of sex robots (eg, for patients with contact disorders, patients with deviant sexual behavior, and patients with different sexual disorders) complement the few considerations about sex robots that have been existing in the scientific discourse so far. The participants in the quantitative study were to a large extent already familiar with sex robots (77%), but there were also therapists who stated that they had not heard or read anything about them yet. This is mainly because of the fact that sex robots are a very young field of research, and experiences in the therapeutic context have not been available yet.

#### Skepticism Versus Openness Toward Sex Robots

On the one hand, this study shows that therapists and physicians are generally skeptical toward sex robots. The existence of sex robots was not rated as very positive. On the other hand, only few respondents stated that the use of sex robots was not conceivable for them and almost half of all respondents could imagine recommending sex robots for therapy. The fact that therapists initially adopted a critical and cautious attitude toward the *introduction* of a new technology for therapeutic purposes was also known in the context of other electronic mental (e-mental) health implementations (eg, telephone or online therapy) [34]. In addition, sex robots are socially controversially discussed and have new fully automated options with artificial intelligence. On the basis of this background, the study shows that the surveyed therapists and physicians are relatively open toward this development.

In the meaning of expectancy-value theory, an attitude (A) toward an object (object O) can be expressed in a function of beliefs (B) toward this object and the evaluations (E) of these expectations. We would like to use this theoretical thinking to better understand the ambivalent attitude of therapists between skepticism and openness. The results of this study provide an insight into the beliefs and evaluations of therapists. It was shown that there are various ethical concerns. These beliefs influence the attitude toward sex robots. For example, it became clear that female therapists and physicians expressed more ethical concerns about sex robots in this survey and rated them more negatively than male therapists. We were able to determine that the ethical evaluation of sex robots strongly depends on whether they are classified as sexual aids, along other sex toys (and their use is normalized), or whether they are understood as a different category, namely, not as objects used *for* sex, but as *social actors* with whom one has sex. The results also show that not only the subjective definition of sex robots and ethical concerns influence the view of therapeutic benefit, but also expectations of the quality of human-robot sexuality. Furthermore, the attitudes toward sex robots as a therapeutic tool were very heterogeneous (see *Relationship Between Attitude and Sociodemographic Data*).

### Comparison With Prior Work

#### Gender Differences

A comparison with existing research results reveals interesting similarities and differences between the attitudes of therapists and the general population toward sex robots. Both the study of the general population and of the therapists indicated significant gender differences. Women rated sex robots more critically than men. Scheutz and Arnolds [25] assume that different judgments about the appropriateness between male and female participants in their survey could come from market and media forces that specifically address heterosexual men as customers and users. Another explanation could lie in the fact that heteronormative ideas of male hegemony are mirrored in the design of current sex robots. Especially in this context, representatives of the radical feminist extensively argue that promoting the development of sex robots reveals a compulsive attitude toward women’s bodies [35]. In addition, Sullins [36] argues that sex robots “contribute to a negative body image.” In the qualitative study, it became clear that sex therapists attach great importance to the physical design of sex robots when it comes to using them for therapeutic purposes. However, they clearly distinguish therapeutic robots from *pornographic sex robots*. Moreover, they advocate that sex robots should be available in different body shapes to promote a realistic and healthy body image. Kubes [37] assumes that the development of sex robots offers a great potential for reducing stereotypes and promoting diversity but current trends in sex robotics, however, do not explore these possibilities. In this study, it became clear that the therapists interviewed took a gender role-conform perspective as shown by the fact that a distinction was made between *male* and *female* sexuality. Moreover, therapists assumed that male rather than female patients could benefit from sex robots. Despite critical attitudes of women themselves and the gender-conform position previously described, possible benefits for female patients were also discussed.

#### Caution Among Therapists

With regard to the possible benefit of sex robots as a therapeutic tool, the general population [26] was more open-minded than the therapists surveyed in this study. Therapists were even more reluctant when it came to recommending sex robots in therapy. This shows a hesitant attitude toward taking responsibility for the use of sex robots in a therapeutic context. On the one hand, this reluctance could be related to the experiences of the therapists. Some therapists evaluate the development of sex robots in line with internet and pornography and mainly observe the negative effects of internet sex addiction in their daily practice. On the other hand, technical and ethical questions regarding treatment are not clarified, which can lead to uncertainty and restraint. A differentiated evaluation of the results in this context shows that not all therapists and physicians have this restraint. Psychologists and sex therapists were more critical concerning the recommendation of sex robots as a treatment tool than physicians. This may be associated with treatment-related considerations; especially, the majority of psychodynamic psychotherapists in this survey will probably also reflect the recommendation to patients from the perspective of transference and countertransference phenomena. Therapists could also ask themselves what bonding needs or experiences of early childhood could be projected onto sex robots by their patients. In the therapeutic context, the question of which early object relationships are represented by the robot may be also interesting. For psychoanalytically oriented therapists, the rule of abstinence may play a role as well when it comes to recommending sex robots in therapy. In contrast to systemically trained therapists, psychodynamically trained therapists in these interviews generally spoke out against a direct recommendation of sex toys in sex therapy. These considerations are supported by studies in which psychodynamic therapists were more critical of new media such as internet interventions than, for example, cognitive behavioral therapists [38].

#### Patients With Deviant Sexual Behavior

With regard to the treatment of pedophile patients, the results showed the opposite picture compared with attitudes in the general population. Although the general population is strongly against the use of sex robots in this context [25], it is controversially discussed by the therapists surveyed in this study. In this context, the consideration was expressed that the use of child sex robots could lead to the prevention of actual children’s abuse. Similar thoughts have already been discussed in pornography research. However, studies have concluded that violent pornography is more likely to increase aggressiveness and therefore has no cathartic effects [39]. The considerations to live out sexual violence and sexual abuse with robots also lead to the question whether there are limits to how a robot should be handled. However, this is a question for the research of robot ethics [40].

#### Differences in Age

In the context of age effects, it would be obvious to assume that the younger generation’s knowledge and openness to technology should also manifest itself in different attitudes toward sex robots. In line with this expectation, the survey found significant differences between younger and older therapists. Younger therapists were actually more open to the topic. Other studies in the field of e-mental health applications also found similar age differences between therapists, for example, in their attitudes toward the internet and online therapy [41].

#### Affinity to Technology

Other studies also showed that the negative evaluation of robots is generally a factor that influences attitudes toward sex robots [28]. In this study, no association with attitudes toward sex robots in relation to the evaluation of negative consequences of technology (TA-EG subscale) was found.

#### Personality Traits

Consistent with our findings that personality traits have no influence on respondents’ attitudes toward sex robots, Szczuka et al [27] also found in their study that personality traits have no effect on the purchase of sex robots.

### Limitations

There are some methodological limitations to this study. Owing to the small sample size, the results of this study need to be interpreted with caution. For this reason, the results are not representative. Comparable online survey studies with psychotherapists, however, show even lower response rates than ours [42]. One could assume that especially therapists interested in technology, who have a positive attitude toward robots, took part in this study. On the other hand, it also needs to be considered that mainly participants with a negative attitude might have participated. In this survey, we could not find any bias, as we got both positive and negative opinion patterns.

Another limitation of this study is that not all respondents shared the same level of knowledge about sex robots. The provision of a stimulus in the questionnaire such as images or film clips to define sex robots makes it possible to survey the same level of knowledge. In this survey, we refrained from using this kind of stimulus as the state of research is still so young that there is hardly any illustrative material available. In addition, media representations of intimate human-robot relationships show stereotypical gender roles and heteronormativity [21].

In spite of the small sample, the investigations provide first exploratory results.

### Implications for Research and Practice

Further research on a larger sample of therapists is necessary to gain a more differentiated picture of the therapeutic potential. Similar to other authors before us, we conclude that ethical responsibility in the digital age cannot be perceived as a *critical distance* from technological development but is effective when sexual scientists play an active role in shaping a technology that can be used to promote sexual health, nonviolence, and sexual diversity [5]. The topic should also be integrated into the training of sex therapists to form opinions beyond media images and to point out therapeutic options. Instead of criticizing only dystopian visions of harmful sex robots, it is recommended to develop robots with positive effects on sexual education, sexual therapy, sexual counseling, and sexual well-being for interested groups. In future research, the different applications of robotic sex (eg, hardware robots and software robots) should be investigated in a differentiated way. The therapists’ experiences with expert knowledge in robot technology and/or robot therapy should be included. The use of robots as a future tool in sex therapy still leaves many moral, ethical, and treatment-related questions unresolved, which need further research and evaluation.

## Appendix

Multimedia Appendix 1 Interview Guide.

## Abbreviations

e-mental: electronic mental
ICD: International Statistical Classification of Diseases and Related Health Problems
TA-EG: The Questionnaire on technical affinity-attitude towards and handling of electronic devices
VR: virtual reality
