# Supplementary material for: The Attitudes of Therapists and Physicians on the Use of Sex Robots in Sexual Therapy: Online Survey and Interview Study
Source: J Med Internet Res. 2019 Aug 20;21(8):e13853. doi: 10.2196/13853 (PMC6719485; doi:10.2196/13853)
Supplement: Multimedia Appendix 1 [file jmir_v21i8e13853_app1.pdf]

Interview Guide

1. What do you know about sex robots and how did you inform yourself about sex robots (e.g. psychotherapy training, media, own fantasies, etc.)?

2. What do Sex robots need to have to be more widely used in a therapeutic context? How should sex robots look like? Which further features or functions do you consider relevant for the therapeutical use of sex robots ?

3. If you recommend sex robots to patients, which information would such a recommendation contain in a therapeutic context? (In the case of no recommendation, ask if other technical "tools" can be suggested and their difference to sex robots)

4. In our survey, some therapists suggested using sex robots for pedophile patients who are not allowed to live out their sexuality. What is your view on this consideration?

5. In our survey, it became clear that besides the general imaginability of sex robots use in therapy, there are also ethical concerns. How can these contradictions be resolved?

6. How can we - as therapists – contribute to the development of sex robots?

7. Do you have any other thoughts or suggestions on the subject that you consider as relevant?
